# Protic Ionic Liquids for Lignin Extraction—A Lignin Characterization Study

**DOI:** 10.3390/ijms19020428

**Published:** 2018-01-31

**Authors:** Ezinne C. Achinivu

**Affiliations:** Ionic Liquids & Electrolytes for Energy Technologies (ILEET) Laboratory, Department of Chemical & Biomolecular Engineering, North Carolina State University, Raleigh, NC 27695, USA; ecachini@ncsu.edu or eachinivu@gmail.com; Tel.: +33-6-2588-4264

**Keywords:** lignin, lignocellulose, biomass, bioproducts, biopolymer, lignin functionality, aromatic structure, protic ionic liquids

## Abstract

Protic ionic liquids (PILs) have been established as effective solvents for the selective extraction and recovery of lignin from lignocellulosic biomass. In this study, we utilize extensive analytical techniques to characterize the PIL-extracted lignins to (1) expand on the physical/chemical structure, and to (2) develop a better understanding of the mechanism behind the lignin dissolution process. The PIL-lignins were characterized using elemental and FT-IR analyses, alongside molecular weight distribution and chemical modeling via MM2. For the more ionic pyrrolidinium acetate ([Pyrr][Ac]), there is an increase in the fragmentation of lignin, resulting in lignin with a smaller average molecular weight and a more uniform dispersity. This lends better understanding to previous findings indicating that higher ionicity in a PIL leads to increased lignin extraction.

## 1. Introduction

Lignin is the second most abundant polymer in nature, and has the potential to be a renewable source of high value chemicals for use in several significant applications [1,2,3,4]. Due to its unique functionality, lignin can be directly transformed to aromatic specialty and fine chemicals that can function as a basic building block chemical—performing similarly to petroleum-based analogues (benzene, xylene, toluene) [3]. Lignin can also be employed in composite materials to improve the performance of other polymers. Several advantageous properties can be realized (antioxidant, anti-bacterial, anti-UV, etc.) by incorporating small amounts of lignin with other polymers [4]. The potentials are limitless for the creation of lignin-based bioproducts (polyols, epoxy resins, feedstock adhesives, and for carbon fiber), and this could be a significant source of revenue generation that will sustain the bioeconomy for years to come [4,5,6].

These opportunities for lignin valorization have motivated significant research activity concerning the extraction of lignin from biomass and its subsequent utilization. However, the significant variation in the quality of lignin on the market, along with difficulties in degrading it selectively, have made it undervalued and underdeveloped. Depending on the biomass source utilized along with the lignin extraction method (pretreatment), various lignins can be liberated that may have different properties [7,8]. Typical pretreatment methods, such as physical (milling and grinding), physicochemical (steam pretreatment/auto hydrolysis, hydrothermolysis, and wet oxidation), chemical (alkali, dilute acid, oxidizing agents, organic solvents, and ionic liquids), biological, electrical, or a combination of these, have been studied for the extraction of lignin from biomass [8,9,10,11,12,13,14,15,16,17,18,19,20]. Therefore, there is a need for the selective extraction of high quality lignin (purity and functionality) with physiochemical properties of interest for certain applications.

**Figure 1:**
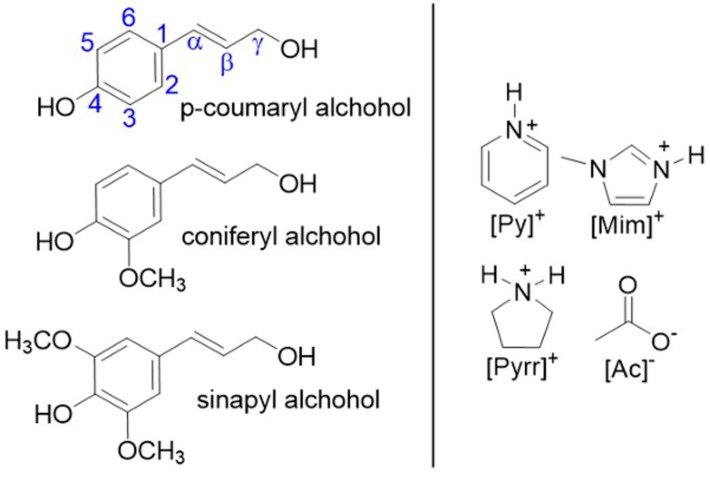
(**left**) The basic units of lignin; *p*-coumaryl (hydroxyphenyl), coniferyl (guaiacyl), and sinapyl (syringyl) alcohol, and (**right**) the PIL ions and their abbreviations: pyridinium [Py]^+^, 1-methylimidazolium [Mim]^+^, pyrrolidinium [Pyrr]^+^and acetate [Ac]^−^.

The use of protic ionic liquids (PILs) for lignin extraction is an emergent area of research that has been demonstrated to have relatively high lignin extraction efficiency [21,22,23,24,25,26,27,28]. PILs are a unique class of ionic liquids (also known as liquid salts) that are produced via a simple acid–base neutralization, such as the combination of organic acids and amine bases [21], and thus, are relatively cost competitive chemicals [21] (note: one or more of the ions can also be inorganic). Several research groups have surveyed this technique due to is ability to selectively extract large amounts of lignin from biomass (leaving most of the cellulosic portion unaffected) [21], along with its potential for recyclability [21]. In our previous work, three PILs were surveyed for their lignin extraction efficiency (Figure 1) [21]. The pyrrolidinium acetate [Pyrr][Ac]-PIL emerged as the most efficient for lignin extraction (%e = 76), due to its high ionicity [21]. Several factors have been postulated to understand the lignin extraction process, however, the mechanism for lignin dissolution and regeneration in the PILs is not fully understood. Additionally, the properties of the lignin recovered (PIL-lignin) needs to be evaluated for use in potential applications.

Therefore, this study utilizes several characterization techniques to elucidate the structure of the extracted lignin from lignocellulosic biomass, as well as to give further insight into the interactions between the PIL ions and lignin that support high lignin solubility. The PIL-lignin extracts were characterized using elemental and FT-IR analyses, along with an evaluation of the molecular weight distribution (MWD), and finally, chemical modeling via MM2. Lignin from different plant sources are usually classified according to the abundance of their basic phenylpropane units: guaiacyl (G), syringyl (S), and 4-hydroxyphenyl (H) (Figure 1) [29,30,31,32,33,34,35,36]. The characteristics of the lignin obtained depend on both the source of the lignin and intensity of the delignification process [29,30,31,32,33,34,35,36]. These model compounds can be used along with molecular modeling and FTIR analyses to confirm the lignin purity, structure, and functionality. (FTIR) is a simple non-destructive technique, which can be utilized to obtain quick and accurate information about the structure of lignin [37]. The molecular weight (MW) of lignin is a fundamental property that influences the valorization of lignin, and can be readily measured using gel permeation chromatography (GPC) [38,39,40,41]. The effect of the solvent used to regenerate the lignin was also considered.

For these assessments, the impact of the PIL type on lignin was determined using kraft lignin (K. lignin) as a standard. The components of the cation and anion of PILs can be easily fine-tuned to adjust their physicochemical properties [21,42]. This can affect their implementation for the extraction and recovery of lignin from biomass. Additionally, the lignin extracted from corn stover (CS) using [Pyrr][Ac] was recovered an analyzed. These analyses will help to improve understanding of the mechanism behind lignin dissolution and extraction from biomass. 

Researchers have studied the impact of pyridinium carboxylate based PILs on lignin. These studies reported interesting properties (pertaining to increased purity and changes in MW), nevertheless, the studies focused solely on kraft lignin for experimentation [27,28]. Kraft lignin is an already modified lignin extract that is recovered from the pulp and paper industry [17,18]; therefore, those results cannot be utilized as the sole indication of the PIL effectiveness for lignin extraction. Analyses of real biomass must be considered for the ultimate application of these PILs in biomass processing. Furthermore, these results focus on pyridinium-based PILs, although, previous results indicate that these PILs are inferior to the pyrrolidinium based PILs for lignin extraction [21].

## 2. Results and Discussion

### 2.1. Lignin Fragmentation (MWD)

The MW and dispersity of any biopolymer (like lignin) are important properties that have been shown to affect the performance and consequent application of lignin in the development of high value bioproducts [38,42]. Therefore, the ability to control or tailor these properties is of high importance to end users of lignin for various applications [38,40,41]. Analyses of the average MW of lignin can also provide insight into the fragmentation pattern of lignin during PIL dissolution/extraction, which can be used to develop further understanding of the mechanisms that promote lignin dissolution in PILs. For this study, all lignin samples were acetylated, dissolved in THF, and subsequently, analyzed using a GPC for their MWD [30,38,39,40,41]. Investigations were performed on the kraft lignin samples recovered after PIL dissolution (PIL-K. lignin), as well as the lignin samples that were recovered after extraction from corn stover (PIL-CS lignin). 

For the PIL-K. lignin-derived samples, the results show that the average MWs are generally lower than that of the original kraft lignin sample. This indicates that the “PIL dissolution process” further depolymerized the lignin, thereby yielding smaller fragments of lignin (Table 1). Amongst the PILs surveyed, there is also a trend towards lower MWs with increasing ionicity. The more ionic [Pyrr][Ac]-PIL corresponds to a greater extent of proton transfer between the acid/base pair [21]—resulting in a mixture with a relatively high ionic character. Ionic liquids have been of interest due their tunable physical properties, such as their densities, viscosities, and conductivities, which can influence their solvation power [43]. Previous research results also revealed that the increased ionicity also corresponded to a more effective lignin extraction [21]. While there were other factors that contributed to the lignin extraction (such as the partial dissolution of the xylan—a hemicellulose) [21], it is worth noting that this high ionic media also resulted in the recovery of lignin with increased fragmentation, thereby producing lower MW lignin.

MW: [Py][Ac]-K. lignin > [Mim][Ac]-K. lignin > [Pyrr][Ac]-K. lignin

The outcomes of this study also show that more ionic [Pyrr][Ac]-PIL produced a lignin extract with a lower dispersity. For these extracts, the lignin fragmentation pattern is more ordered, and yields lignin with a relatively high uniformity (even compared to the original K. lignin). These observations are in agreement with recently reported results where researchers have found that the use of PILs for lignin dissolution results in a “reduction in polydispersity and the average molecular weight” [27]. However, our results show that not all the PILs have this performance. We observe that the lower ionicity PILs ([Py][Ac] and [Mim][Ac]) both have increased dispersities (Table 1). 

For the CS-extracted lignin, where there is better control over the starting material (since kraft lignin is a lignin extract with an already modified chemical structure), these research outcomes are greatly highlighted. The MWs and dispersity of the lignin recovered directly from corn stover ([Pyrr][Ac]-CS lignin) are lower than for any of the kraft lignin samples. This indicates that the regenerated lignin had a more homogenous distribution (Table 1), which could be a property of high interest for applications were purity, uniformity and quality control is of significance [38,40,41]. 

**Figure 2:**
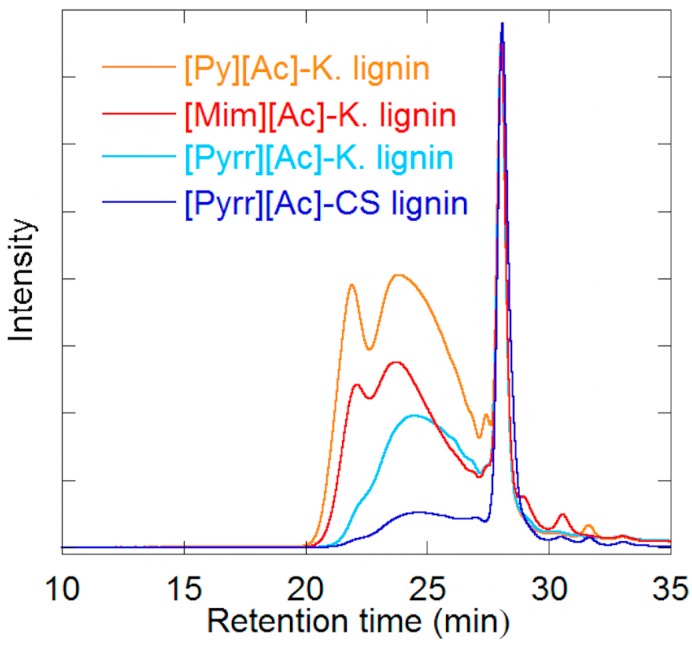
Size exclusion chromatograph of acetylated Kraft lignin recovered from PIL dissolution and CS lignin from the [Pyrr][Ac]-PIL.

These outcomes are supported by the SEC chromatograms from which the MWs were calculated. Figure 2 shows that in the [Pyrr][Ac]-CS lignin, with lower dispersity, there is a reduction of the broad peak at t < 27 min for larger MW lignins, while the intensity of the peak at t ~ 28 min for lower MW lignin is heightened. This confirms that the [Pyrr][Ac]-PIL is more suitable for uniform lignin fragmentation. It is important to also note that for the [Pyrr][Ac]-CS lignin, the acetylated lignin samples are not completely dissolved in THF as this lignin is closer to native lignin, and is not easily dissolved in typical organic solvents (unlike kraft lignin). This has also been observed in other PIL-extracted lignins [30]. However, the conclusions described in the previous paragraph remain valid, as the data from the [Pyrr][Ac]-K. lignin chromatogram supports the trends observed.

Based on these results, the ionicity of the PIL is an important factor for yielding lower MW lignin with a low dispersity. The PIL dissolution process is a purely physical process that involves the coordination of several weak molecular-level interactions to enhance lignin dissolution. Ionic media (like the PILs) have the ability to stabilize charged biopolymer intermediates via electrostatic/coulombic forces, which could increase the PIL’s solvation power [44]. These ionic interactions (between the PIL and lignin extracts) are likely one of the mechanisms that promote “uniform lignin fragmentation”. In the past, focus has been on hydrogen bonding as the main molecular-level interaction between ionic liquids and biopolymers [21]. While hydrogen bonding plays a significant role in lignin dissolution, ionic attraction through coulombic forces might also have an important function in the dissolution process, and could also influence the properties of the resultant lignin. 

While high MW lignin can offer improved performance in a material’s mechanical properties [38], lower MW lignin is a model feedstock for adhesive manufacturing [38]. Additionally, researchers have found that the lower MW fractions have greater solubility for polymeric solutions, due to a higher content of functional groups, such as phenolic hydroxyl groups and methoxyl groups [40,41,45]. The low MW fractions were also dispersed more uniformly in polymeric composites, yielding materials with reproducible mechanical properties [41].

### 2.2. Elemental Analysis

This section discusses the elemental analysis and degree of unsaturation (DOU) of the PIL-K. Lignin-derived samples, together with PIL-CS lignin-derived samples for the [Pyrr][Ac]-PIL. (Table 2) The degree of unsaturation is calculated with the simplified equation below based on the empirical formula [46]:

(1) $$Ring+\pi Bonds\;\left(DOU\right)=C-\frac{H}{2}-\frac{X}{2}+\frac{N}{2}+1$$

where *C* = number of carbons, *H* = number of hydrogens, *X* = number of halogens and *N* = number of nitrogens; oxygen and other divalent atoms do not contribute to the degree of unsaturation. The molecular masses for *C*, *H*, and *N* used are 12.0107, 1.00794, and 14.0067 g mol^−1^, respectively.

According to the results, there is an insignificant change in the DOU for the PIL-K. lignin samples. Therefore, the PIL dissolution process does not cause a reduction in double bonds or DOUs, and the recovered lignin maintains its original functionality. At the conditions used for lignin extraction (90 °C, 24 h), the primary interaction between the PILs and the lignin are weak molecular-level bonds, such as hydrogen bonding, therefore, a reduction in DOU should not be observed [21]. For the [Pyrr][Ac]-CS lignin, however, the DOU is slightly lower. This is possibly due to the increased fragmentation of lignin observed with this PIL (confirmed with the MWD analyses—above). Although these PILs are not strong enough to break double bonds, an increased fragmentation of lignin could lead to a relatively lower amount of double bonds in the lignin extract—hence the lower DOU. 

The elemental analyses also show a slight increase in the N amount for the PIL-recovered lignins. This is possibly due to the incorporation of small amounts of the PIL with the lignin during dissolution. The main source of N to the system is from the PIL’s cation (amine bases). For the [Pyrr][Ac]-PIL, the pyrrolidine (cation source) is a cyclic aliphatic ammonium with no DOUs. The presence of small amounts of this reagent can cause an effective reduction in the DOU. Nevertheless, these results confirm the purity and functionality of the lignin, and no elemental sulfur (or inorganic ions) was introduced into the system—as is the case for some lignin extracts [17].

### 2.3. Lignin FT-IR Analyses

In-depth FT-IR analysis was used to characterize the absorption bands for representative functional groups in the lignin extracts (Figure S1). Table 3 lists the peaks observed, as well as their assignments using previously described biomass samples [31,32,33,34,35,36,37]. To understand the impact of PIL dissolution, the IR spectra of three representative samples are analyzed (K. lignin, PIL-K. lignin, PIL-CS lignin) (Figure S1). For these analyses, emphasis was placed on the [Py][Ac] PIL, due to the characteristic peaks that enabled easy identification of this PIL in the IR spectra. The [Py][Ac] PIL arises from the aromatic base pyridine, which has a sharp peak at ~1600 cm^−1^ for aromatic skeleton vibrations, (Figures S1 and S2), as well as a characteristic peak for the C=O stretching of the carbonyl groups at ~1700 cm^−1^ for the acetate anion (acid source) (Figures S1 and S2).

The IR spectra show slight changes in the measured absorbance in the regions described above. This suggests that small amounts of the PIL might remain incorporated with the lignin samples after recovery. The maximum PIL recovery (from previous work) was 98%, which was observed during the use of [Py][Ac] PIL [21]. Additionally, observing changes in the characteristic peaks for the anion and cation indicate that both ions contribute to the lignin dissolution for these PILs. Nevertheless, this amount (~2%) is likely insignificant, due to the fact that the lignin also contributes to the IR peaks in this region (Table 3). The lignin recovered was also thoroughly washed by water, and the relative ratio of N to C is very low for the lignin extracts (Table 2).

**Figure 3:**
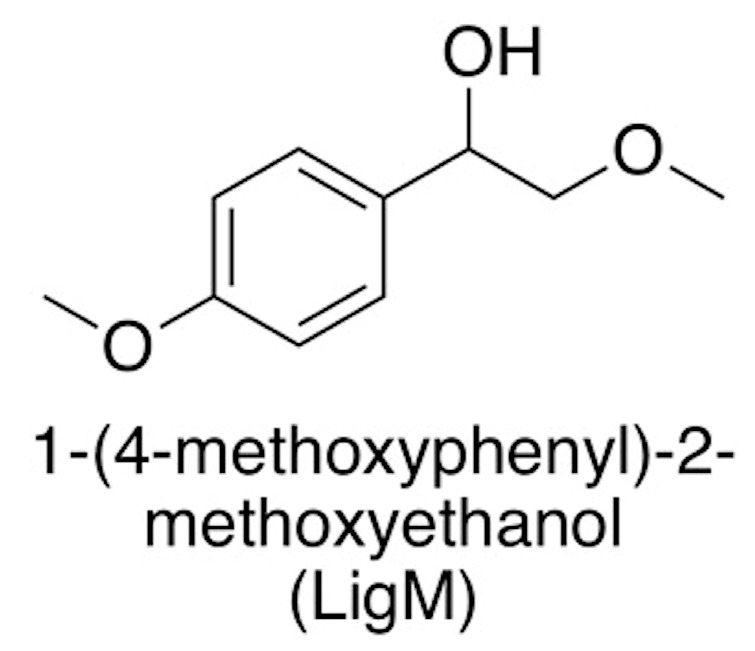
Chemical structure of the 1-(4-methoxyphenyl)-2-methoxyethanol (LigOH) (β-*O*-4 linkage) chosen as the model of lignin to investigate the interaction between lignin and PILs at the molecular level.

For biomass characterizations, assignments were made based on knowledge of the sources of each material. Kraft lignin is extracted form pinewood, a softwood lignin, which mainly consists of guaiacyl units [34]. Therefore, the characteristic peaks for syringyl absorption at 1325 cm^−1^, as well as the C–H out of plane in position 2 and 6 (syringyl units) at 825 cm^−1^, are not present in the kraft lignin samples (Figure 3). Additionally, the peak at 875 cm^−1^ for C–H out-of-plane vibrations in position 2, 5, and 6 of the guaiacyl units is only observed in the kraft lignin samples. Lastly, the relative intensity of the peak unique to guaiacyl due to the C–O stretching of the guaiacyl unit, found at 1250 cm^−1^, is much higher in kraft lignin than that of corn stover lignin—due to the higher ratio of guaiacyl to syringyl units in softwood lignin [34,35,36,37,38,39,40,41,42,43,44,45,46]. Corn Stover, a grass species, has the three types of lignin components present (guaiacyl and syringyl lignin, and *p*-hydroxyphenyl) [35]. Guaiacyl and syringyl peaks are typically easier to characterize, and have significant absorbance peaks that enable the clear conclusions derived from the FT-IR spectra (Figure 3 and Table 3). These analyses confirm that the recovered lignin maintains its functionality, leading to a highly chemically viable and thermally stable product (Figure S3).

### 2.4. Energy Minimization (MM2 Analyses)

The components of lignin are connected with several linkages, such as β-*O*-4, 5-5, β-5, 4-*O*-5, β-1, dibenzodioxocin, and β–β linkages (Figure 1). The β-*O*-4 linkage is dominant, consisting of more than 50% of the linkage between the monolignol structures of lignin [47]. Therefore, 1-(4-methoxyphenyl)-2-methoxyethanol (LigM) (β-*O*-4 linkage) (Figure 3) was chosen as a model of lignin to investigate the interactions between lignin and the PIL ions at the molecular level. This model has been utilized in the past for rapid molecular modeling/computational analyses of lignin during ionic liquid dissolution [47,48].

**Figure 4:**
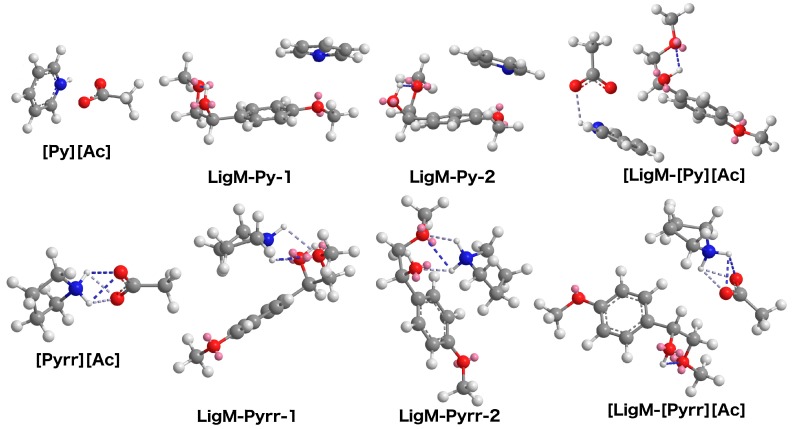
Energy minimization for LigM-PIL for the [Py][Ac]-PIL (LigM-[Py][Ac] and LigM-Py) and [Pyrr][Ac]-PIL (LigM-[Pyrr][Ac] and LigM-Pyrr).

Analyses were performed on the LigM structure, the LigM–LigM dimer (to identify the corresponding interactions found in the lignin monomers), as well as the PIL-LigM structures (for the [Py][Ac] and [Pyrr][Ac]-PILs) (Figure 4 and Figure S4). The resulting structures show intra- and inter-molecular H-bonding between the terminal –OH groups in the LigM molecules (Figure S4). The LigM–LigM2 structure with H-bonding had the lowest energy state. (Figure S4) In this configuration, the simulations also depict that the aromatic groups of the LigM molecules are aligned in a manner that indicate the presence of π–π interactions between the two rings (Figure S2). These comparatively weak interactions amongst the lignin subunits (along with covalent bonds), can contribute to the intractability and reasonably high thermal stability observed in lignin. Therefore, a suitable lignin solvent should be able to participate in these interactions in order to dissolve large amount of lignin from biomass. 

For the [Py][Ac] PIL, the aromatic pyridinium cation also aligns with the benzene rings of LigM—indicating π–π interactions. This orientation also depicts the PIL ions hydrogen bonding, as the N from the amine cation interacts with the O from the anion—an orientation that the PIL ions do not have without the LigM structure (Figure 4). The pyridine base is also able to have π–π interactions with the benzene rings of LigM, explaining the high kraft lignin solubility observed in this reagent (Figure 4) [21].

The PIL of interest, however, is the [Pyrr][Ac]-PIL (which has been shown to extract large amounts of lignin from biomass). This has been attributed to the high ionicity of the PILs, as well as, the hydrogen-bonding network established in these PILs. The simulations, however, do not provide much information about the interactions between these PIL ions and the LigM molecule (Figure 4). The minimal energy state of the PIL ions and the LigM molecule is the same when they are apart. The pyrrolidine base (unlike the pyridine base above) interacts with the –OH groups in the LigM molecules, and has no interactions with the benzene rings. This reagent has a low kraft lignin solubility [21], indicating that the π–π interactions between the aromatic groups are important for lignin dissolution in the aromatic–amine PILs. These analyses provide further information into the factors that contribute to lignin dissolution. However, more extensive simulations with multiple ions for the PILs and a three-dimensional lignin model could be beneficial for delineating the mechanism behind lignin dissolution on a macromolecular level [47]. 

### 2.5. Effect of the Solvent Type on Lignin Regeneration

To end the study, a special consideration for the regeneration of lignin using different solvents is discussed. Typically, PIL recycling and subsequent lignin recovery is accomplished using a simple distillation apparatus [21]. This process was optimized to minimize intensive energy consumption, therefore, (2 to 5)% *w*/*w* of the PIL was unrecovered after the distillation [21]. There is also the potential of extracting small amounts of mono-/oligo-saccharides, since the PILs can also dissolve small amounts of xylan (a hemicellulose) along with the lignin [21]. To solve this problem, the lignin solids need to be washed/regenerated using a suitable solvent that can remove any residual PILs, as well as dissolved sugars.

Research has shown that the type of solvent used to recover the lignin (and other biopolymers) typically governs, to some extent, the resulting biopolymer’s properties, which could be important for lignin applications [41]. The solvent used has to be insoluble in lignin, while also being soluble in the PIL. Most molecular solvents are only able to dissolve small amounts of kraft lignin, indicating their suitability for recovering lignin. The solubility of lignin in ACTN, ACN, EtAc, Et_2_O, HEX, HAc, EtOH, and water (Figure S5) was determined gravimetrically, and was confirmed using UV–vis analysis by re-dissolving the recovered lignin residue in 0.1 M NaOH, and using a kraft lignin calibration curve to determine the amount of lignin present (Figure S6).

The lignin recovered remained chemically unchanged. The FT-IR spectra of the kraft lignin recovered are largely identical for all the solvents used (Figure S7). However, there is a peak around 1700 cm^−1^ that is intensified in the acetone-lignin and acetic acid-lignin samples. This peak is due to the C=O carbonyl functional group found in both solvents mentioned. Residual amounts of the solvent might be remaining within the lignin, which give rise to this peak observed. Changes in the physical appearance of the lignin are noted, which is most likely due to differences in the particle size of the lignin extract upon recovery (Figure S7). Amongst the solvents tested, the polar protic solvents (acetic acid, ethanol, water) are the most suitable for this process, due to their ability to dissolve the PILs (Figure S6). Although they dissolve the highest amount of lignin, this amount is relatively small when compared to that of the PILs. The solubility for lignin in water, for example, is 18 g/L, which is about 0.018% *w*/*w* (in comparison, the PILs can dissolve >50% *w*/*w* of lignin).

**Figure 5:**
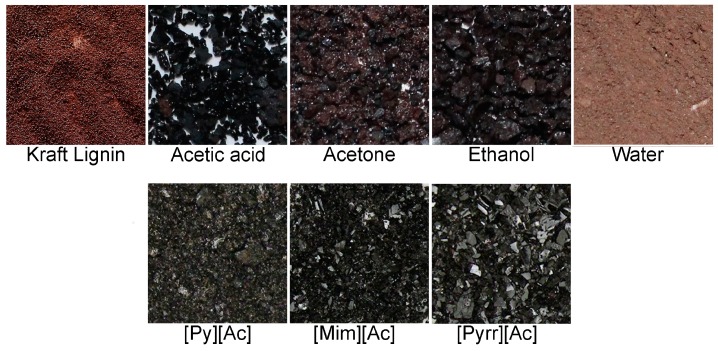
Images (10×) of kraft lignin recovered from solvent and PIL dissolution after heating at 90 °C and 24 h.

**Figure 6:**
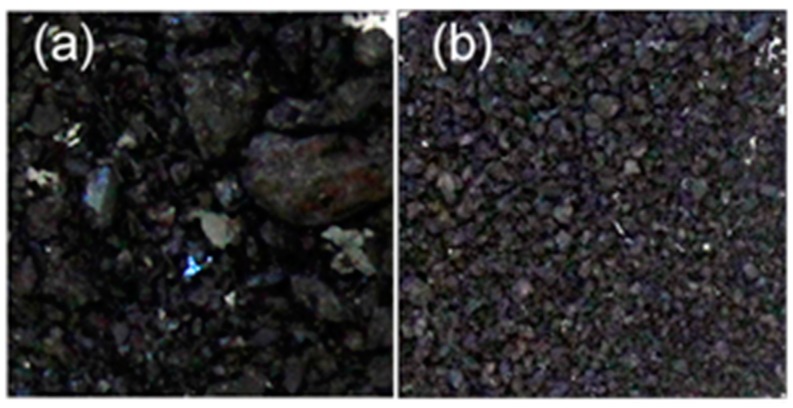
Images (10×) of lignin showing the lignin extracted from CS using the [Pyrr][Ac]-PILs: (**a**) the solids recovered after the PIL removal, and (**b**) solids after passing the solids form (**a**) through a water wash step.

These tests led to the selection of water for the lignin recovery. Water had the lowest K. lignin solubility, and is also more abundant and environmentally benign when compared to the other two solvents of interest (above). The kraft lignin recovered after water dissolution has relatively fine particles, which might be ideal for recovering homogenous lignin solids (Figure 5). This could positively affect the dispersity of the lignin as well. This technique was then used to recover the [Pyrr][Ac]-CS lignin (Figure 6). The result, as expected, indicates that more uniform lignin solids are being recovered, although they are greater in size than for the water-kraft lignin. The recovered lignin could be ground and size partitioned, to further reduce its particle size and improve homogeneity.

## 3. Materials and Methods

### 3.1. Lignin Extraction and Recovery from Biomass (Corn Stover)

Neat PILs were used to extract lignin from corn stover (CS) as described in our previous publication [21]. The materials used and the procedures adopted were exactly the same as those described in the published literature [21]. Following the lignin extraction, the PIL-lignin liquor was recovered and subsequently separated into two streams (lignin and PIL) via vacuum distillation. The residual solids (CS lignin), recovered after distillation, were then passed through a water wash to remove any residual PIL or dissolved sugars. Kraft lignin samples were also dissolved in each PIL, and then recovered as described above. These lignin extracts (PIL-K. lignin and PIL-CS lignin) were then characterized and analyzed with the following techniques (below).

### 3.2. Molecular Weight Determination (Lignin Acetylation)

The lignin samples were fully dried under vacuum at 40 °C. Approximately 25 mg of each of the lignin samples was dissolved in pyridine (1.5 mL) followed by acetic anhydride (1.5 mL). The acetylation was carried out overnight in the dark for 24 h. The resulting solutions were then poured in an excess (50 mL) of ethanol and centrifuged/washed three times. The solvent was removed using a rotary evaporator. After this, the lignin samples were fully dried under vacuum at 40 °C. The obtained acetylated samples were dissolved in tetrahydrofuran (THF). The molecular weight was then characterized by a GPC using size exclusion chromatography (SEC). 

### 3.3. Molecular Weight Determination (Gel Permeation Chromatography (GPC))

The gel permeation chromatography (GPC) system consisted of a Shimadzu LC- 20AD pump, a Shimadzu CTO-20A oven (35 °C) equipped with two Waters Styragel column (HR1 & HR5E, molecular range 0~4 M), a Shimadzu SPD-20A UV–vis detector (dual wavelength, 235 nm and 254 nm were used for all experiment) and a Shimadzu RID-10A reflective index detector, which were used to analysis the samples. The THF was used as the mobile phase with a flow rate of 0.7 mL/min. A calibration curve was created based on 12 polystyrenes (PSS ReadyCal-Kit Poly) from MW of 266 to 2.52 × 106 g/mol. Data collection and processing were done by Shimadzu LC solution software. 

### 3.4. Elemental Analyses 

*C*, *H*, and *N* analysis were performed for the lignin extracts by Atlantic Microlab, Inc., and used to calculate empirical formula and degree of unsaturation.

### 3.5. FT-IR Spectroscopy

IR spectroscopy was performed using a PerkinElmer Spectrum 2000 FT-IR spectrophotometer. Spectra were recorded as a thin film between KRS-5 plates, in the range 400–4000 cm^−1^, and were accumulated for 32 scans at a resolution of 4 cm^−1^. The spectra were baseline corrected and processed using the available software.

### 3.6. MM2 Analyses

A two-dimensional structure was depicted by Chemdraw (Cambridge Software, MA, USA) and was converted to a three-dimensional structure by Chem 3D (Cambridge Software) followed by a structural optimization (lowest energy) using MM2 (molecular mechanics 2). The MM2 parameters are based on information provided by Dr. Allinger, with minor modifications by Dr. Ponder [49,50,51,52]. The following color scheme is used to interpret the images generated from the model: red (oxygen), blue (nitrogen), dark grey (carbon) light grey (hydrogen), pink (electron lone pairs), and dashed lines (H-bonds). H-bonds with a less than ideal geometry are displayed with a blue tint. The intensity of the color increases as the bond becomes less ideal. The behavior of the PIL ions along with a lignin model compound (LigM) was observed using molecular mechanics model (MM2-Chem 3D Pro). By utilizing the inbuilt force field functions, energy minimizations were carried out for each PIL-LigM combination to minimize the bond stretching energy of each molecule. This helped to identify the molecular-level interactions that are present in the PILs with high ionicity, and clarify the influence of H-bonding on the PILs’ properties and lignin dissolution capacity.

### 3.7. Lignin Solubility and Regeneration in Various Solvents

The suitability of several solvents for lignin regeneration was tested. First, the solubility of lignin in these solvents—ACTN (acetone), ACN (acetonitrile), EtAc (ethyl acetate), Et_2_O (diethyl ether), HEX (hexane), HAc (acetic acid), EtOH (ethanol), and water (Figure S6)—was determined gravimetrically using the procedure previously described [21]. The solubility was also confirmed using UV–vis analysis by re-dissolving the recovered lignin residue in 0.1 M NaOH, and using a kraft lignin calibration curve to determine the amount of lignin present (Figure S6). The residual lignin was then recovered for analyses.

### 3.8. Imaging 

Photographic images of up to 10× magnification were also taken using a Canon Rebel T3i EOS 600D 18.0 MP Digital SLR Camera to observe the lignin morphology after dissolution and regeneration in PILs and certain solvents.

## 4. Conclusions

This study provides deeper insight into the characteristics and properties of the lignin extracts that are recovered after interacting with the PILs during lignin dissolution/extraction. The [Pyrr][Ac]-PIL, which has a greater ionicity, favors the fragmentation of lignin and results in more homogenous particles (low dispersity). This is suitable for applications where uniformity and quality control are of high significance. Elemental and FT-IR analysis both confirm the functionality and purity of the recovered lignin extracts. However, small amounts of the PIL might remain incorporated with the lignin (even after washing with solvents). These results show that the cation interacts directly with the lignin molecules, while also interacting with the anion. Both interactions, working together, differentiate the ionic solvent (PIL) that is able to dissolve large amounts of lignin. Finally, various solvents can be used to wash lignin after dissolution, which control the morphology of the resulting fibers.

## Figures and Tables

**Table 1:** Weight-average (*M*_w_) and number-average (*M*_n_) MWs (g mol^−1^) and dispersity (*M*_w_/*M*_n_) of the kraft lignin recovered from PIL dissolution and the lignin extracted from corn stover (CS) using the [Pyrr][Ac]-PIL.

PIL	*M*_n_	*M*_w_	*M*_w_/*M*_n_
K. Lignin (Indulin AT)	1600	6500	4.06
[Py][Ac]-K. lignin	830	4983	6.00
[Mim][Ac]-K. lignin	646	3919	6.06
[Pyrr][Ac]-K. lignin	528	1797	3.40
[Pyrr][Ac]-CS lignin	330	900	2.73

**Table 2:** Elemental analysis DOU of the recovered lignins.

PIL	*C* (%)	*H* (%)	*N* (%)	Degree of Unsaturation
Kraft lignin	62.2	6.1	0.9	3
[Py][Ac]-K. lignin	63.7	5.8	1.4	3
[Mim][Ac]-K. lignin	63.5	6.0	3.7	3
[Pyrr][Ac]-K. lignin	64.5	7.2	4.0	3
[Pyrr][Ac]-CS lignin	52.1	10.3	10.1	1
EF-CS	43.0	5.7	0.9	2

**Table 3:** Absorption peak assignment in FT-IR spectra of Kraft lignin recovered from PIL dissolution and the lignin extracted from CS using the [Py][Ac] PIL [31,32,33,34,35,36].

Approximate Band (cm^−1^)	Assignment
3350–3400	O–H stretching in hydroxyl groups
2975, 2925	C–H stretching in methyl and methylene groups, C–H stretching aromatic methoxyl groups
2850	–CH_2_– symmetry stretching in methyl and methylene groups
1700–1725	C=O stretching in unconjugated ketone, carbonyl, and ester groups
1650 ^a^	C=O stretching in conjugated ketone p-subst. Aryl ketones
1600 ^b^	Aromatic skeleton vibrations plus C=O stretching; S > G
1500–1525	Stretching of aromatic skeleton; G > S
1450	Aromatic skeletal vibrations C–H deformations (asymmetry in methyl group –CH_3_– and –CH_2_–) O–CH_3_ in-plane deformations.
1375	Aliphatic C–H stretching in methyl and phenol OH
1325 ^a^	(C–O of syringyl ring) S unit plus G unit condensed (G unit bound via position 5)
1250	C–O stretching of guaiacyl unit
1225	C–C plus C–O plus C=O stretching
1125 ^a^	Typical of S unit; also secondary alcohol and C=O stretch
1050–1025 ^c^	C–O of primary alcohol, C–O–C ether stretch, guaiacyl C–H
875 ^c^	C–H out of-plane vibrations in position 2, 5 and 6 of the guaiacyl units
825 ^a^	C–H out of plane in position 2 and 6 (syringyl units)

Peaks highlighted are significant to ^a^ CS-lignin, ^b^ [Py][Ac]-treated lignins, ^c^ kraft lignin.

## Supplementary Materials

The following are available online at http://www.mdpi.com/1422-0067/19/2/428/s1.

## Abbreviations

PIL	protic ionic liquid
[Py][Ac]	pyridinium acetate
[Mim][Ac]	1-methylimidazolium acetate
[Pyrr][Ac]	pyrrolidinium acetate
MWD	molecular weight distribution
DOU	degree of unsaturation
CS	corn stover
K. lignin	kraft lignin
MM2	molecular mechanics 2
%e	lignin extraction efficiency
