# Supplementary material for: Protic Ionic Liquids for Lignin Extraction—A Lignin Characterization Study
Source: Int J Mol Sci. 2018 Jan 31;19(2):428. doi: 10.3390/ijms19020428 (PMC5855650; doi:10.3390/ijms19020428)
Supplement: Supplementary file 1 [file ijms-19-00428-s001.pdf]

Supplementary Information
Protic Ionic Liquids for Lignin Extraction—A Lignin Characterization Study

Ezinne C. Achinivu *
Ionic Liquids & Electrolytes for Energy Technologies (ILEET) Laboratory, Department of Chemical & Biomolecular Engineering, North Carolina State University, Raleigh, NC 27695, United States; ecachini@ncsu.edu

* Correspondence: ecachini@ncsu.edu

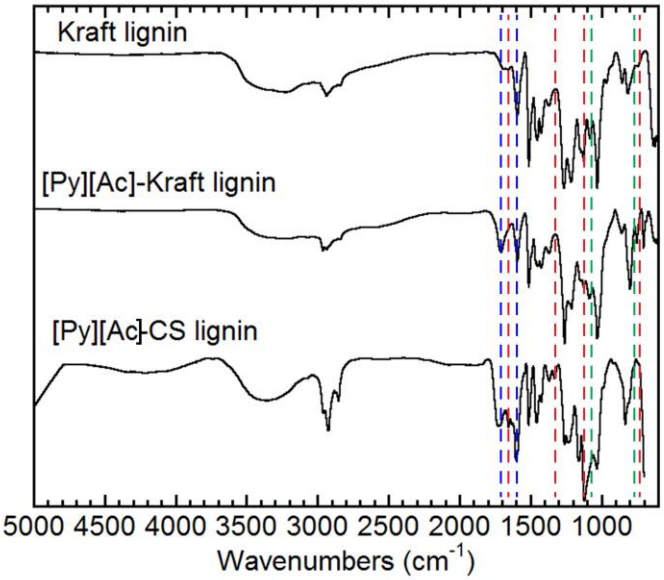

Figure S1. FT-IR spectra for Kraft lignin recovered from PIL dissolution and the lignin extracted from CS using the [Pyrr][Ac] PIL

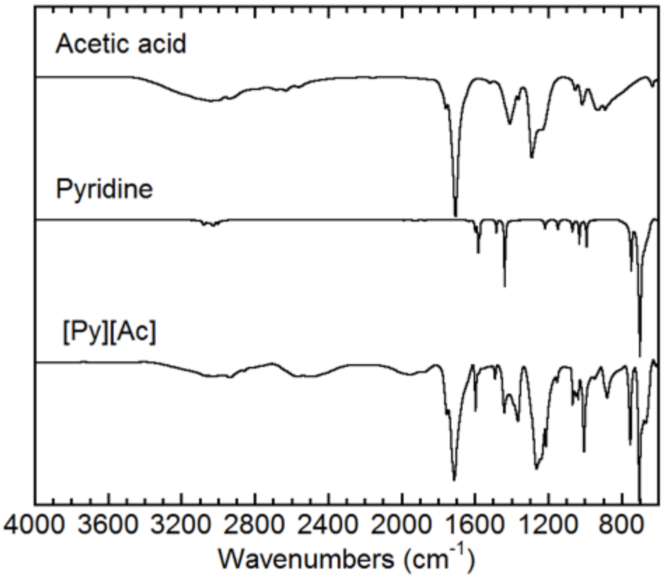

Figure S2. FT-IR spectra for the [Py][Ac] PIL and the reagents used to synthesize it (pyridine and acetic acid).

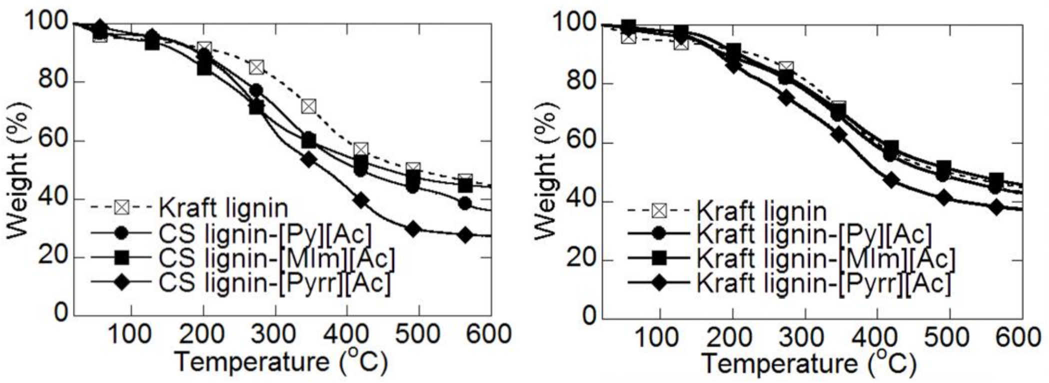

Figure S3. (left) Variable-temperature TGA heating traces of Kraft lignin and the recovered lignin from CS after PIL treatment, and (right) Variable-temperature TGA heating traces of original (Kraft lignin) and recovered lignin from PIL dissolution. (Reproduced from previous publication) [1].

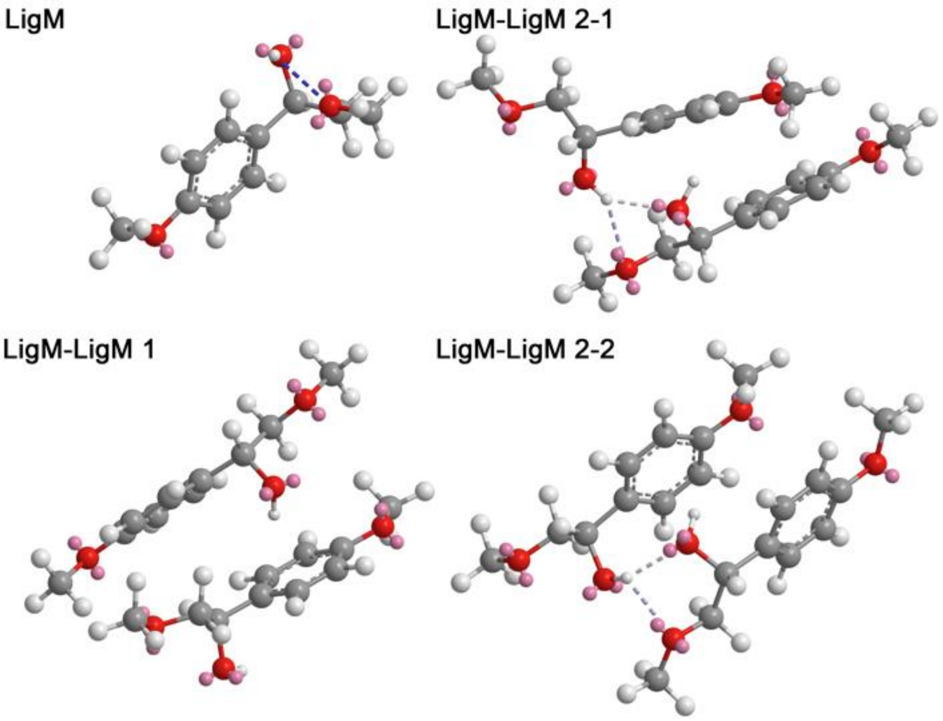

Figure S4. Energy minimization of the lignin model compound (LigM), LigM-LigM.

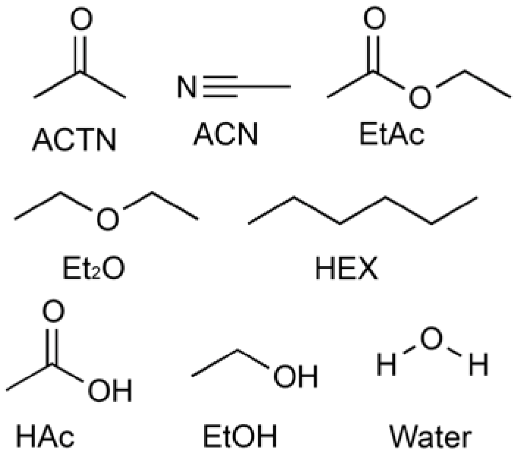

Figure S5. Solvents used for lignin dissolution and regeneration study.

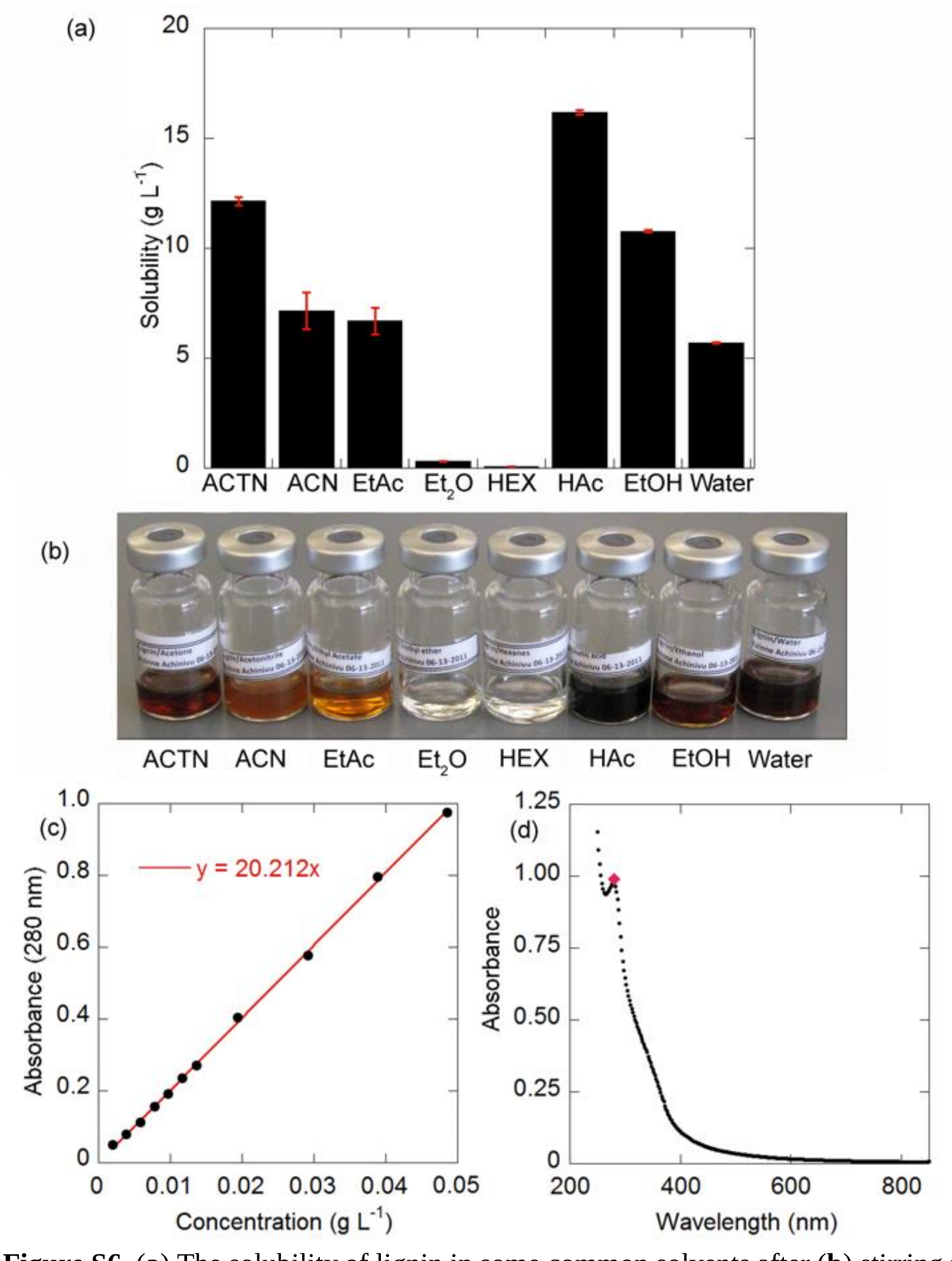

Figure S6. (**a**) The solubility of lignin in some common solvents after (**b**) stirring for 24 h at 90 °C, recovering the supernatant, solvent evaporation, and re-dissolution in 0.1M NaOH as determined by: (**c**) the Kraft lignin calibration curve at (**d**) the wavelength of maximum absorbance.

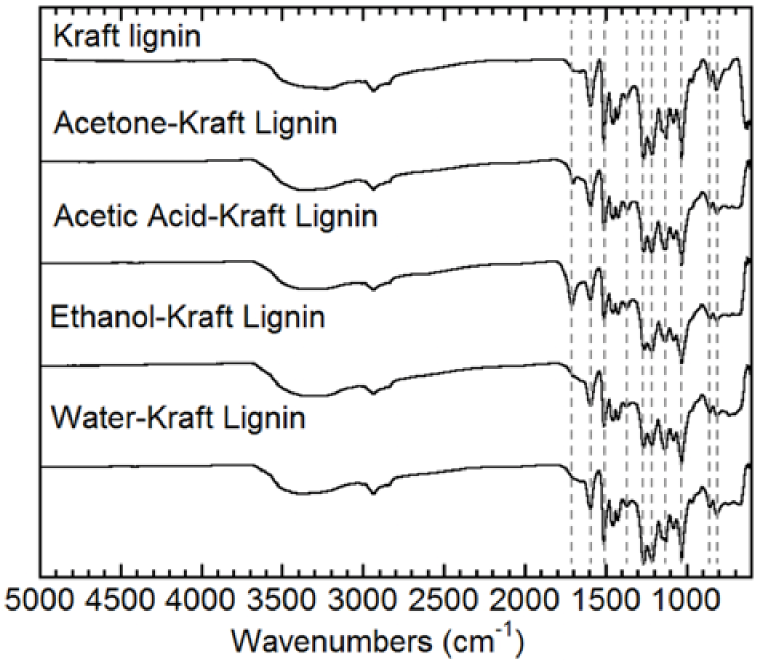

Figure S7. FTIR spectra of lignin regenerated from solvent dissolution after heating at 90 °C and 24 h.

Reference:

1. Achinivu, E. C.; Howard, R. M.; Li, G.; Gracz, H.; Henderson, W. A. Lignin extraction from biomass with protic ionic liquids. Green Chem. 2014, 16, 1114–1119, doi:10.1039/C3GC42306A.
